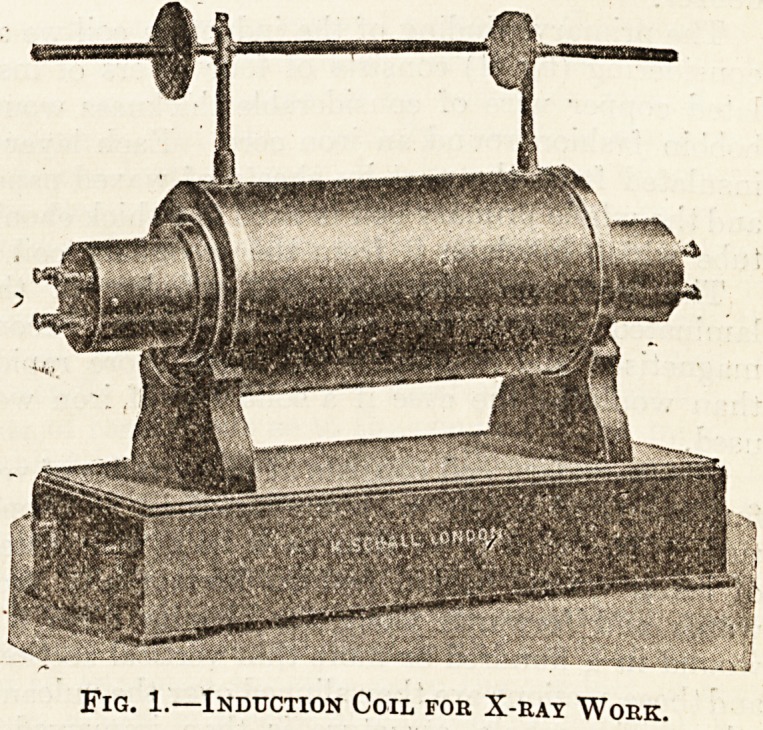# The X-Rays

**Published:** 1912-03-30

**Authors:** Alfred C. Norman

**Affiliations:** House Surgeon at Sunderland and Durham County Eye Infirmary, Sunderland.


### ELECTRICITY IN MODERN MEDICINE.*

### IX.—The X-Rays.

By ALFRED C. NORMAN, M.D. Edin., House Surgeon at Sunderland and Durham County Eye Infirmary, Sunderland.

Before considering the *x*-rays there is still a little more to be said about the "Pantostat" motor generator. The motor will do work up to one-eighth-horse power, and there is a slot on the axle to which a flexible steel shaft can be clamped for driving surgical drills and trephines and the various apparatus used in vibratory massage. Less than an ampère of current is required to drive the motor, so that it is quite safe to connect the Pantostat to any lamp-fitting or wall-plug. This machine is a veritable electro-medical installation in itself, and its comparatively low cost brings electro-therapeutics within the scope of even the smallest and poorest of hospitals, provided they have an electric lighting supply.

Fig. 2, page 430, of The Hospital for January 27 illustrates the general arrangement of a Pantostat motor generator. It can be obtained for use either on an alternating- or on a continuous-current supply. The appearance of the machine is the same in either case, the only difference being in the construction of the motor, which is rather more complicated when made for an alternating-current supply. For use on a continuous-current supply this machine costs about £23, for an alternating-current supply it is rather more expensive. It is quite possible to carry it about from ward to ward, but it is more satisfactory to obtain it mounted on a light trolley with rubber castors.

### THE X-RAYS.

On April 30, 1895, Professor Röntgen, of Würzburg, while investigating the cathode rays from a Crookes tube excited by an induction coil, discovered an entirely new form of radiation now known to all the world as the Röntgen or *x*-rays.

Röntgen was working in a dark room with his Crookes vacuum tube completely enveloped in a black cardboard box, so that no visible light could escape into the room, when he observed that barium platinocyanide became highly luminous as soon as it was brought within a metre or so of the cardboard box. Obviously some form of radiation had penetrated cardboard perfectly opaque to ordinary light and had excited the barium salt to fluorescence. *Röntgen had discovered the* x-*rays* and, unlike most great discoveries, the tremendous importance of this was at once recognised by the whole scientific world.

Sir William Crookes, Lennard, and Hittorf must all have been many times on the verge of making this same discovery during their laborious investigations of the cathode rays, and it is food for thought that these scientists—seeking knowledge as they were in realms from which, at most, they hoped to glean some facts of theoretical interest—should have paved the way to a discovery of such enormous practical importance.

### The Production of X-Rays.

Radiography has evolved beyond the stage of empiricism and is now an exact science conforming to definite laws by which constant and uniform results can invariably be obtained. Good work can be done with comparatively simple apparatus, but the user must thoroughly understand every part of it; the writer therefore advises a beginner to start with an absolute minimum and gradually to add to his stock of apparatus as increasing knowledge brings home to him the necessity for higher refinements of technique. In this way he will avoid the confusion likely to arise if he attempt to control so

Fig. 1.—Induction Coil for X-ray Work.

many varying factors at the outset, and he will acquire as he goes along a thorough understanding of each piece of apparatus, of its possibilities and limitations, and of its exact influence upon the working of the installation as a whole.

To consider all the various ways of producing *x*-rays and to discuss even briefly the many types of transformer, interrupter, switchboard, and the like, would take us far beyond the scope of this article and would serve no useful purpose. The writer therefore proposes to describe in some detail a simple and comparatively inexpensive *x*-ray installation from which it is not difficult to get really first-class results. He has used this installation in three different hospitals (having himself installed it in two of these) and has never once found the apparatus at fault.

* Previous articles appeared on Nov. 11 and 25, Dec. 9 and 30, Jan. 13 and 27, Feb. 17, and March 9.

The installation consists of the following apparatus: A vacuum tube and induction coil (fig. 1) to produce currents of sufficient voltage to overcome the high resistance of the vacuum tube; a switchboard to control the current passing through the primary winding of the induction coil; an interrupter rapidly to make and break the current passing through the primary of the induction coil, as well as a fluorescent screen, tube holder and stand, couch, milliamperemeter, and photographic outfit.

### The Induction Coil.

The resistance of an $x$-ray tube runs into millions of ohms; it is therefore necessary to use a current of very much higher voltage than we have yet had to deal with. This high pressure (or high tension as it is usually termed) current may be produced in various ways, such as by a static machine or by an alternating-current transformer of special design, such as the Gaiffe or Snook pattern, but in this country by far the most satisfactory method is to use an induction coil. An induction coil consists essentially of a primary winding, an iron core, a secondary winding, and, in most instances, a condenser.

The primary winding of the induction coil we are considering (fig. 1) consists of four layers of insulated copper wire of considerable thickness wound bobbin fashion round an iron core. Each layer is insulated from the next by sheets of waxed paper, and the whole primary coil is fixed in a thick ebonite tube which insulates it from the secondary coil.

The iron core consists of a number of thin laminated sheets of soft iron which become magnetised and de-magnetised much more rapidly than would be the case if a solid bar of iron were used.

The secondary coil consists of many miles of very fine insulated copper wire supported on an ebonite tube. The wire is as thin as a hair, and instead of being wound bobbin fashion horizontally backwards and forwards along the ebonite tube, it is wound in a hundred or more thin vertical sections, and these sections are then slipped over the vulcanite tube. The whole structure is then immersed in melted paraffin wax to obtain as perfect insulation as possible and is fixed inside a large ebonite cylinder.

If we took 100 reels of cotton and joined the end of one reel to the beginning of the next and so on until they were all connected in series, so to speak, and if we then slipped all the reels side by side on to a long tube, we should have an arrangement somewhat similar to the sectional winding of a good induction coil, but in an induction coil each section is no thicker than a disc gramophone record.

The advantage of vertical sectional winding is that the sections are more satisfactorily insulated from each other than they would be if they were wound horizontally along the tube, and this is a matter of considerable importance since we are dealing with currents which may reach 250,000 volts.

The condenser consists of a pack of very many flat sheets of tinfoil, each sheet being separated from the next by a layer of mica or of waxed paper. The alternate sheets of tinfoil are so arranged that their ends project from opposite sides of the pack and are there bound together with metal clamps, so that one clamp grips Nos. 1, 3, 5, 7 in the series, while the other grips Nos. 2, 4, 6, 8, and so on. The sheets of tinfoil are thus divided into two sets, the elements of one set being completely insulated by the waxed paper from those of the other, although they are so closely interleaved.

The condenser may be quite separate from the induction coil, but it is usually fitted into a wood box at the base of the coil, its opposite sides being connected by means of the metal clamps with two terminals on the front of the base. We shall see later that the condenser acts as a reservoir for "self-induced" current in the primary coil, thus preventing undue sparking between the contacts of the interrupter, but to attain this the two sides of the condenser must be connected with opposite sides of the interrupter.

When sent out by the makers an induction coil consists of the following separate parts: the primary coil, encased in a stout ebonite tube; the secondary coil, encased in ebonite and supported on a hollow tube; the wood base, consisting of a box (containing the condenser) and two upright supports for the secondary coil; and two discharging pillars. It is a simple matter to put together the apparatus. The secondary coil has to be placed upon its base, the primary coil slipped into the tube which runs through the secondary, and the discharging pillars pushed into sockets provided for them on the secondary coil.

Fig. 1 shows the induction coil ready for use. At each end of the cylinder containing the primary coil there are four terminals, those at the right-hand end being engraved : A-1, 2, 3, 4, and those at the left-hand end: E-1, 2, 3, 4, the former corresponding to the beginnings and the latter to the ends of the four layers of the primary windings. These four layers are quite separate from each other, so that by means of the terminals in question we can connect them together either in series or in parallel, or partly in series and partly in parallel. If we connect them all in series we shall obtain a high resistance and a high self-induction in the primary coil, whereas by connecting them in parallel the resistance and self-induction will be low. Such a coil is said to have a "variable primary" or a "variable self-induction," the significance of which will be explained later. For a mercury interrupter a coil should have a high self-induction, and it is therefore used with all the layers connected in series, but for an electrolytic interrupter it is a great advantage to be able to vary the self-induction while the coil is working, and for this a special switch, known as a "Walter" switch should be obtained. The connections of a Walter switch are rather complicated, but once it is fitted to the base of the coil or to the switchboard it is only necessary to turn the handle to connect the layers of the primary coil in series, or in parallel, or in series-parallel, at will.

*(To be continued.)*

## Figures and Tables

**Fig. 1. f1:**